# Biodeterioration agents: Bacterial and fungal diversity dwelling in or on the pre-historic rock-paints of Kabra-pahad, India

**Published:** 2013-09

**Authors:** Jayant Biswas, Kavita Sharma, K.K. Harris, Yogita Rajput

**Affiliations:** 1 National Cave Research and Protection Organization, Central Laboratory, Raipur, C.G., India; 2 Arts and Commerce Girls college, Raipur, C.G, India; 3 Department of Zoology, Govt’ DB Girl's PG College, Raipur, C.G., India

**Keywords:** Cultural heritage, Rock-paint, Bio-deterioration, microbial attacks, Rock weathered

## Abstract

**Background and Objectives:**

In the last few decades, losses of our cultural heritage due to biodeteriorationare beinghighly recognized. From museum objects to rock monuments, the microbial biodeterioration agents are found to be the most destructive. Possibilities for proper preservative measure(s) are always more when it is only a monument, statue, museum article, or pre-historic art in any small subterranean cave. Nevertheless, preservation/protection of the footprints occupying a big area, lying scattered in a very negligible manner requires safeguard against several deterioration factors; right from various physical, chemical and biological agents which are indeed interrelated to each other.

**Materials and Methods:**

In the present study, some microbial communities possibly responsible for deteriorating the rocks of Kabra-pahad, where the most famous pre-historic rock paints of India prevail have been identified. The diversity of fungi and bacteria present in the stone crust of the infected areas has been studied by employing standard laboratory methods.

**Results:**

The cultivated cultures confirmed total fifteen fungal species, among which *Aspergillus* group were the most dominant. Among bacteria, total 80 numbers of colonies were observed that dominated by two major groups; *Micrococcus*.spp and *Staphylococcus* spp.

**Conclusion:**

The pre-historic footprint in the form of rock paints in Kabra-pahad of district Raigarh, Chhattisgarh, India is lying in a very deteriorated manner. In the present study, we have tried to identify few major deteriorating factors that are responsible for such degradation of our existing pre-historic footprints.

## INTRODUCTION

All over the world, the footprints of most of the pre-historic arts are lying scattered in a very baleful manner, awaiting proper care and preservative measures. Among them, the rock paintings are the most valuable part of our ancient cultural heritage, as it has witnessed the presence of prehistoric civilization, their respective sense of creativity and artistic abilities. The rock painting tradition is very old, dating back to prehistoric times and is also considered as one of the fine arts; practiced by early man to decorate their residential vicinity, usually cave shelters. The rock paintings also help us to understand about the contemporary societies, which reflect the pride of that particular nation's cultural heritage by its overall inventory. Thus, the importance for proper protection of such rock paints hardly needs any explanation. Rock deterioration is a result of various physical, chemical and biological factors acting together, which could be synergistic or antagonistic in nature. However, limited attempts have been taken to examine the essential role that the biological agents play in the deterioration of the same (1–7). As per Hueck (8), ‘*any undesirable change in the properties of a material caused by the vital activities of living organisms*’ is biodeterioration. Since long time, deterioration due to several biological agents has been recognized in various ancient rock based monuments, rock paints and other relevant structures in the tropical countries where various environmental factors viz., high temperatures, high relative humidity levels, and heavy rainfall possibly favour the growth and sustenance of a wide variety of microbial communities on their respective surfaces (1). Further, fungi are the most established detrimental microbes among all the microbial communities. In the past few years, around the world it has been realized that the rock paint existing inside caves are the most precious heritages which are suffering from serious fungal attacks. In this regard, the most victimized case of Lascaux cave could not be ignored (9).

Till date, a great variety of species viz.bacteria, algae, lichens, archaea, fungi, mosses and even plants that colonize rocks and/or mural paintings have been well established. Rock being inorganic in characteristic it never supports the growth of fungi in itself, even though it remains permanently wet. But, various supporting organic energy sources in the form of other microbes (algae/bacteria), decaying debris insects and/or bird droppings occurring in the rock surfaces usually serves as food sources for them to grow (10).

## MATERIALS AND METHODS

### Study Site

**Fig. 1:**
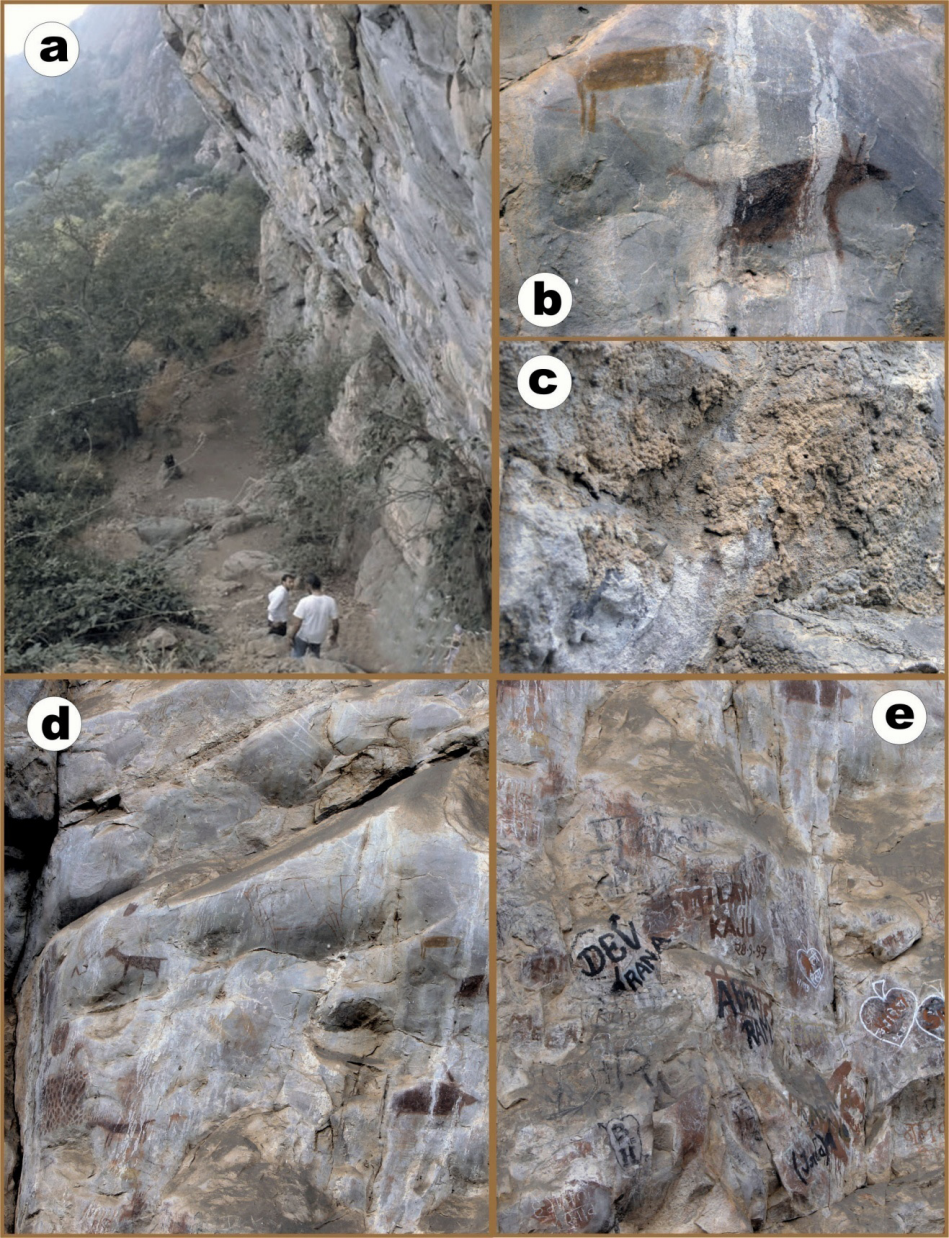
Different views representing the present status of Kabra-pahad a) Side view of slanted rock shelter of KabraPahad b) Deteriorating pre-historic paint c) Close-up view of decayed rock, from where sample was collected d) Seepage from rock joints deteriorating the rock paint e) Notorious activities by the current visitors.

Kabra-pahad (pahad = mountain) (21.50N 83.27E) a huge rock-shelter of about 20-25 m tall and 16-18 m broad, situated at about 16 km away from the Raigarh district, the most developing industrial belt of the state Chhattisgarh, India. This rock-shelter is inclined at an angle, which protects its main wall directly from every environmental stress throughout the year (Fig. 1a). The complete structure is composed of soft sedimentary rock, consisting mainly of a large variety of sand stone (calcareous and siliceous) and limestone which is easily susceptible to weathering. At about 10 m above the base level of this rock-shelter, various painted figures of animals and human-beings of pre-historic era subsists. Besides Kabra-pahad, there are many more such types of sites are exist in this zone where these types of rock paints are also found. Interestingly, most of the sites pertaining to the Rock paints are apparent nearer to some subterranean caves or big rock-shelters (11) which directly suggests that pre-historic people lived in such caves/rock-shelters by forming small societies.

Possibly from the initial stage, due to the soft nature of this sedimentary rock shelter, the figures painted in its wall are in peril. The seepage from the rock cracks is continuously weathering the complete outer surface of this rock-shelter. Thus, the major portions of the painted figures which comes in-way of water flow are also completely deteriorating (Figs. 1b, c, d).

The site is surrounded by dense forest. The temperature and relative humidity were noted with the help of digital make equipment in two specific spots:

- the foothill of Kabra-pahad : Relative humidity − 70%; Temperature −32°C,
- the open plain plateau region at about 500m away from Kabra-Pahad: Relative humidity − 20%; Temperature −38°C

The geophysical parameters, adjacent to the Kabra-pahad area (site-1) were found to be notably differed from the plain plateau area (site-2).

### Identification of mcrobial communities

Patches of large rock weathered areas are clearly apparent due to long timed water seepage from the joints in the wall of Kabra-pahad. The study samples in the form of metamorphosed powdery sediments of weathered rock were collected from the wall adjacent to the painted area. Samples were scrapped off with the help of sterilized scalpel and collected in a sterile plastic airtight vial for its microbial analysis. More stress was given for mycological analysis. Pilot survey to detect the bacterial colonization was also attempted in the same sample.

### Fungi isolation

For isolation and identification of fungi, Potato Dextrose Agar (PDA) media were prepared containing chloramphenicol (500 mg/litre) to control the bacterial growth in the same. One gram of the washed sample was inoculated randomly in the PDA figure and three replicates were simultaneously maintained. Following one of the prescribed method (12), the plates were incubated at 28±1°C and examined daily for the growth rates and sporulations, continuously for 7 days. After completion of incubation period, the various isolated fungal colonies were transferred into fresh PDA plates. The procedure was continuously repeated for 5 times to remove the impurities.

Various fruiting structures having taxonomic significance were identified by preparing lactophenol cotton blue *(LCB)* wet mount. Finally, the cultures were sent to National Center of Fungal Taxonomy, Indiafor authenticity.

### Bacterial isolations

The study was carried out by employing dilution figure (13) and direct figure (14) techniques. Further, following the methods suggested by the Bergey's Manual of Systematic Bacteriology (15, 16) bacterial colonies were identified based on morphological identity, biochemical test and their respective physiological activities.

## RESULTS AND DISCUSSION

### Fungal diversity

**Fig. 2:**
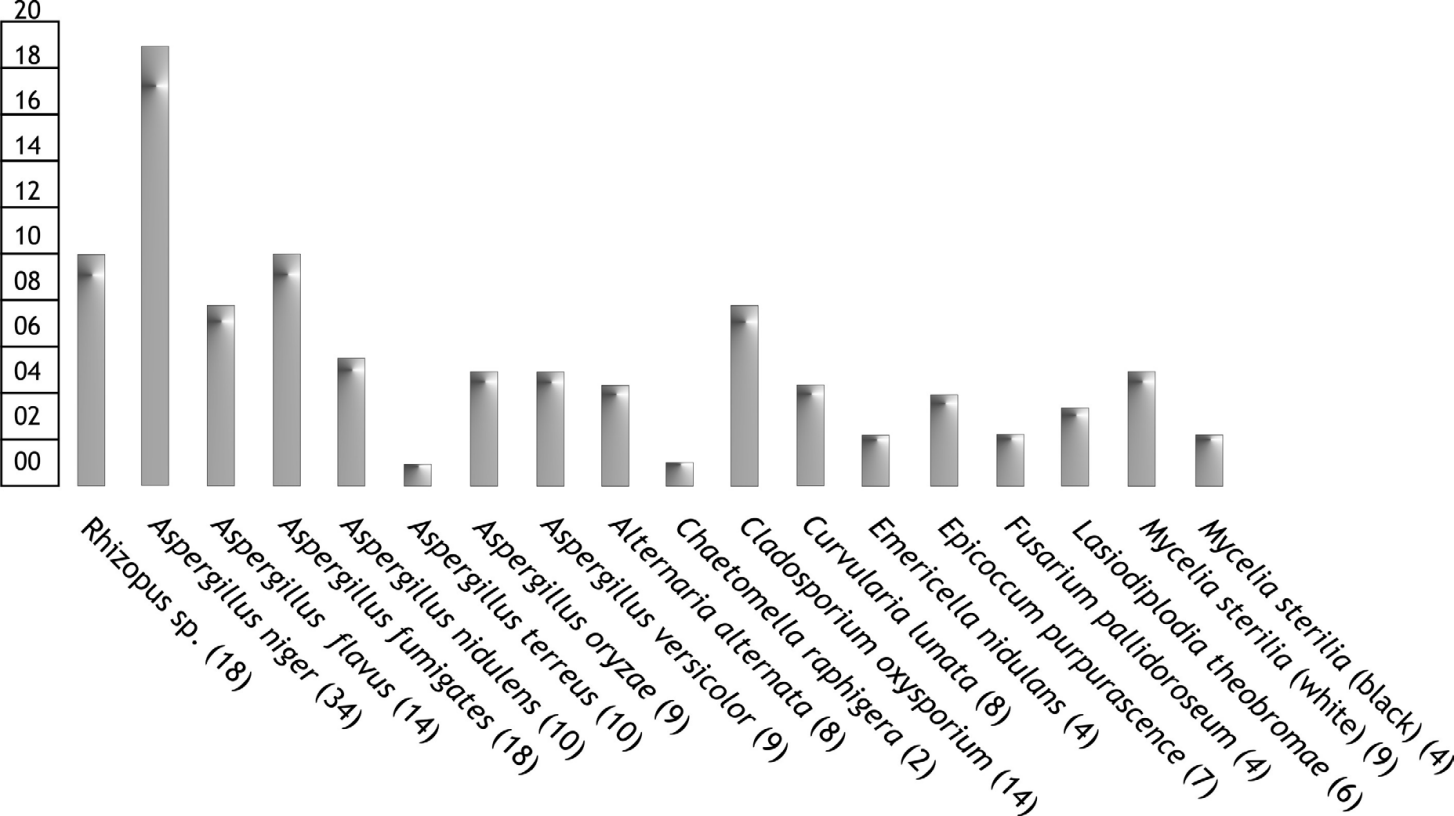
Percentage contribution of Fungal Species in rock decayed sample of Kabra-pahad. Number of colonies observed against each species is also included in the brackets

Obtained results are demonstrated in Fig. 2.

Totally, eighteen fungal species were confirmed from the cultivated cultures (including *2* strains of *Mycelia sterilia)*, among which *Aspergillus* group were the most dominant, reported their presence with seven specific species; *A. niger, A. flavus, A. fumigates, A. nidulens, A. terreus, A. oryzae, A. versicolor*. Most of the obtained *Aspergillus* species were already reported to be the deteriorative in most of the stone monuments and other relevant historic cultural heritages in the tropical region (1, 17). In the present study, *Aspergillusniger* (Anamorphic fungi) was contributed maximum, and is the most destructive component in this concerned. Indeed, most of the fungal species isolated from the weathered stone crust sample were already reported as the biodeteriorative agents for various ancient monuments exist in this part of Central India, (Chhattisgarh) by our associate mycologist (18–21).

Bacterial growths are also vulnerable to painting pigments and its respective underlying rocks. In addition, it also serves as nutrient source for fungal growth on the rock. Total 80 numbers of colonies were observed on agar which was dominated by two major groups; *Micrococcus* and *Staphylococcus*. Bacterial identification at the preliminary stage was done by using probabilistic identification of bacteria (PIB) Win software (22). It is a database developed for the identification of bacteria based on numerical taxonomy. *Micrococcus luteus-4* (ID score- 0.9996) was found to dominate the colony with 40 CFU value, where as 30 and 10 CFU values were revealed against *Micrococcus agilis* (ID score- 1) and *Staphylococcus hyicus* (ID score- 0.99767) respectively. *M. agilis* and *S. hyicus* showed circular configuration and concave elevation hence *M. luteus-4* appeared in the form of irregular configuration but flat elevation. Various bacterial colonies have been isolated from different rock samples, confirming the dominance of Gram-positive organisms. Both the *Micrococcus* ssp.isolated by us are very common soil bacteria for this region, we even recovered it from the deep zones of caves from India (23–25).

## CONCLUSION

The rock paintings are indeed a representation of fine arts; practiced by early man to decorate their ambient vicinity of shelters. Kabra-pahad represents one of them. Unfortunately due to high deterioration it is on the way of losing its importance. The water oozing from the sutures of the rock wall is directly or indirectly weathering the complete rock shelter of Kabra-pahad and ultimately, fleeting our pre-historic footprints. The rate of weathering for any rock depends on various environmental factors existing around it, especially the mineral Vs. water interactions and the inherent property (chemical structure) of the rock itself and the rate of reactivity of the mineral surfaces (26, 27) which in the preliminary stage chemically deteriorate it. Further, the microbial communities accumulate on the same; accelerate the rock decay manifolds, collectively known as ‘bio-weathering’. Unfortunately, for the pre-historic rock paint sites, the preservation strategies for natural decaying processes are usually overlooked during conservation management plan. Universally, the professionalism in conservation of rock paint is very rare. Researches on rock paint usually focus on characterization and dating issues, rather than conservation (5).

As it has already been discussed that the rocks never support the growth of fungal colonies, nevertheless, various environmental factors such as ambient humidity, dampness, climatic exposure, energetic sources with its few inherent parameters supports the establishment of the same in it (28). In the present case, the geophysical parameters, adjacent to the Kabra-pahad area were found to be highly favorable for microbial colonization. The same conditions do not exist at a distance of 500m away from it. It is an established fact that the high relative humidity (>55%) and the dampness of the rock surface promotes the growth of fungi in the rock surfaces. Further, Kabra-pahad is standing in a relatively tilted position which never allows direct pressure of rain, due to which regular washing of superficial microbial colonies from it surface could not get possible. Nevertheless, the process of water leaching from the wall junctions is obviously making a paste with aeroflora, epifauna, bird/bat droppings, bacterial colonization etc. which ultimately supports the proliferation of the fungi; the most deteriorating agent.

The fungal species; *Aspergillus niger*, which dominantly occurred in the sample with some other fungi is already known to produce such enzymes, which were enriched with various organic acids like gluconic, citric, and oxalic acids that can solubilize the powdered stone and chelate various minerals in a rich glucose medium (29, 30). However, a systematic study of the chemical alterations in the weathered rock is a pre-requisite to assess the actual role of the identified microbial diversity in the rock wall of Kabra pahad.

During last few decades, universally the rapid growth in industrialization has obviously altered the composition of the entire atmosphere, resulting in rapid deterioration of the existing materials. Today the environmental pollution is a major contributing factor for fungal growth (31) and rock erosion too (32). In addition, due to the global warming effect the possibilities of increasingthe fungal colonization in Kabra-pahad could not be ignored.

Finally, the protection of such pre-historic sitesfrom the modern artists (visitors) is also an essential need, who usually leaves their footprints in the same wall to remember their visits (Fig. 1e). Does it not represent also an example of Biodeterioration?
